# Biofilm-mediated resistance to berberine in *Escherichia coli*

**DOI:** 10.3389/fcimb.2025.1565714

**Published:** 2025-07-17

**Authors:** Yingfang Wang, Ruinan Zhang, Pengfei Wang, Wenlu Zhang, Zhongjie Li, Xinyue Pang, Fangfang Huang, Sensen Wang, Xingnuo Liu, He Zhang

**Affiliations:** ^1^ Henan University of Science and Technology, School of Basic Medical Science and Forensic Medicine, Luoyang, Henan, China; ^2^ Henan University of Science and Technology, School of Hospital, Luoyang, Henan, China; ^3^ Henan University of Science and Technology, School of Medical Technology and Engineering, Luoyang, Henan, China; ^4^ Henan University of Science and Technology, School of Affiliated First Hospital, Luoyang, Henan, China

**Keywords:** *Escherichia coli*, berberine, drug resistance-related genes, biofilm, csgD gene

## Abstract

**Objective:**

To investigate the mechanism of biofilm-mediated resistance to berberine in *Escherichia coli*.

**Methods:**

The resistance of berberine against *E. coli* was induced by 1/2 MIC (minimum inhibitory concentration). Biofilm formation was detected by crystal violet staining. The mRNA level was detected by RT-qPCR, and the gene *csgD* was determined. the *csgD*-overexpressed strain was constructed. We measured the MIC of berberine against *E. coli*, as well as biofilm formation and the expression of mRNA.

**Results:**

The MIC after berberine induction was more than 32 times than the MIC before induction. the biofilm was significantly increased at 24, 48 and 72 hours (*p*<0.01) after berberine induction. In addition, the amount of biofilm production at 24, 48 and 72 hours was 1.3, 1.51 and 1.98 times after berberine induction than that before induction, respectively. The expression of *csgD* gene was significantly increased (*p*=0.016) after induction compared with that before induction. the MIC of *csgD-*overexpressed strain was about 5.8 times that before induction. The expression of *csgD* gene was significantly increased (*p*=0.016), which was 5.8 times higher than that before induction. The MIC of *csgD-*overexpressed strain was 100 μg/mL. Biofilm formation in csgD-overexpressed strain was 2.9 times higher than that of the control. The expression of biofilm-related genes, *bcsA*, *luxS* and *csgD*, was 45, 22.5 and 1628 times higher than that of the control, respectively.

**Conclusion:**

Berberine might increase biofilm formation by inducing the expression of *csgD* gene, which might result in drug resistance in *E. coli*.

## 1 Introduction

Escherichia coli (*E. coli*), a conditional pathogen inhabiting the human intestinal tract, is associated with a wide range of infectious diseases (Riley, 2020; Pakbin et al., 2021; Zhang et al., 2022). According to World Health Organization (WHO) data from 2023, approximately 150 million cases of urinary tract infections (UTIs) occur annually, with 70%–95% attributed to *E. coli*. Globally, 280 million cases of gastrointestinal infections are reported each year, among which diarrheagenic *E. coli* pathotypes (e.g., ETEC, EPEC, and STEC) account for 20%–40%, resulting in 380,000 deaths annually due to E. coli-related diarrhea. Additionally, bloodstream infections (BSIs) exhibit an annual incidence of 1.3 million cases, with *E. coli* being the predominant Gram-negative pathogen responsible for 25%–30% of BSI cases, and mortality rates reaching 15%–35% for antibiotic-resistant strains (World Health Organization (WHO), 2023).

In recent years, the extensive use and misuse of antibiotics have led to a growing crisis of antimicrobial resistance (AMR) in Escherichia coli (*E. coli*), posing a significant challenge to global public health. The World Health Organization (WHO) identifies E. coli as one of the primary pathogens driving the AMR crisis, with the emergence of multidrug-resistant (MDR) strains rendering many conventional antibiotics substantially less effective or even obsolete (World Health Organization (WHO), 2020). The rapid spread of *E. coli*, combined with its propensity for multidrug resistance, biofilm formation, and remarkable capacity to acquire antibiotic resistance genes through horizontal gene transfer (HGT), has further exacerbated its resistance profile. These factors collectively contribute to the escalating severity of antimicrobial resistance in this pathogen (Modi et al., 2013; Kralicek et al., 2018; Katongole et al., 2020; Uruen et al., 2020).

Escherichia coli (*E. coli*) can form biofilms, structures that not only enhance bacterial resistance to antibiotics but also enable long-term survival in harsh environments, thereby increasing the persistence and recurrence of infections (Penesyan et al., 2021). Biofilms are three-dimensional structures formed by bacteria through the secretion of extracellular polymeric substances (EPS) in specific environments, allowing them to adhere to biological or non-biological surfaces. These biofilms serve as both a survival strategy for bacterial adaptation to environmental conditions and a protective barrier against host immune systems and antibiotic (Markova et al., 2018). The formation of *E. coli* biofilms is a multi-step dynamic process, primarily comprising four stages: initial attachment, microcolony formation, maturation, and dispersal (Ballen et al., 2022). Initial attachment involves bacteria adhering to solid surfaces via surface structures such as flagella or pili, influenced by environmental factors like pH, temperature, and nutrient availability (Marshall and Blainey). During microcolony formation, bacteria proliferate continuously, accumulate EPS, and gradually develop a mature biofilm with a complex three-dimensional architecture. Bacteria within the biofilm communicate via quorum sensing to coordinate biofilm formation and maintenance (Whiteley et al., 2017). Dispersal occurs when bacteria in certain regions of the biofilm re-enter the planktonic state, detach from the biofilm, and spread to new environments, potentially initiating new infections (Rumbaugh and Sauer, 2020).

Biofilm formation significantly enhances E. coli’s antibiotic resistance through multiple mechanisms. Firstly, the biofilm matrix physically impedes antibiotic penetration, reducing their effective concentration. Secondly, bacteria within biofilms exhibit varied metabolic states, with some entering a dormant phase that renders them insensitive to antibiotics. Additionally, bacteria utilize quorum sensing systems to synchronize the expression of antibiotic resistance genes, further amplifying resistance. Finally, the biofilm environment facilitates horizontal gene transfer among bacteria, promoting the spread of antibiotic resistance genes (Vestby et al., 2020; Liu et al., 2024).

The formation of biofilms in Escherichia coli is regulated by multiple genes and regulatory networks. The *csgD* gene, part of the two-component regulatory system for biofilm formation in *E. coli*, controls the synthesis of curli fimbriae and cellulose (Yan et al., 2023). The *luxS* gene in *E. coli* regulates biofilm formation by modulating quorum sensing (QS) and metabolic pathways, synthesizing the universal signaling molecule autoinducer-2 (AI-2) to coordinate bacterial group behaviors (Deng et al., 2022). The *bcsA* gene, a key polysaccharide synthesis gene in *E. coli* biofilms, primarily governs cellulose production. Among these, the *csgD* gene is a pivotal regulatory factor (Sana et al., 2024). The *csgD* gene encodes a transcriptional regulatory protein that activates genes associated with curli fimbriae and cellulose synthesis, thereby promoting biofilm formation (Ogasawara et al., 2011). Upon activation, the CsgD protein encoded by csgD binds to the promoter regions of downstream target genes and regulates the expression of the csgBAC operon, *adrA* gene, and *ycdT* gene (Nasu and Maeda, 2024). The csgBAC operon encodes the major structural proteins of curli fimbriae (CsgA and CsgB) and an assembly protein (CsgC). Curli fimbriae are proteinaceous fibers that mediate bacterial interactions with surfaces and other bacteria, facilitating biofilm formation (Serra et al., 2013). The *adrA* gene encodes a diguanylate cyclase that catalyzes the synthesis of cyclic di-guanosine monophosphate (c-di-GMP), a critical secondary messenger molecule that activates the expression of cellulose synthases such as *BcsA*. Cellulose, a polysaccharide, enhances the stability and mechanical strength of the biofilm matrix (Jenal et al., 2017). The *ycdT* gene encodes a cellulose synthesis-associated protein that further promotes cellulose synthesis and secretion (Limoli et al., 2015). Studies have shown that E. coli strains overexpressing the *csgD* gene exhibit enhanced biofilm formation capacity and antibiotic tolerance. Key mechanisms include: The biofilm matrix physically blocking antibiotic penetration, reducing effective antibiotic concentrations. Bacteria within biofilms existing in heterogeneous metabolic states, with some entering a dormant state that renders them insensitive to antibiotics. Expression of the *csgD* gene being regulated by quorum sensing systems, which coordinate the expression of resistance genes to further amplify antibiotic resistance (Karatan and Watnick, 2009; Neag et al., 2018).

Berberine is an isoquinoline alkaloid widely distributed in various plants, such as Coptis chinensis (huanglian), Phellodendron amurense (huangbai), and Berberis vulgaris (xiaobo) (Ai et al., 2021). As a key component of traditional Chinese medicine (TCM), berberine has been utilized for thousands of years in TCM practices, primarily for treating gastrointestinal infections, inflammatory diseases, and metabolic disorders (Habtemariam, 2020). In recent decades, advancements in modern pharmacological research have unveiled its diverse biological activities, including antimicrobial, anti-inflammatory, antioxidant, antitumor, and hypoglycemic effects (Kumar et al., 2015). In TCM, berberine is predominantly employed to manage gastrointestinal infections (e.g., bacterial dysentery and enteritis) and inflammatory conditions (e.g., oral ulcers and skin infections). Its antimicrobial and anti-inflammatory properties make it a vital therapeutic agent for infectious diseases. Its extensive historical use and robust clinical experience underscore its high safety and efficacy. Investigating the mechanisms by which berberine induces drug resistance can provide scientific insights for optimizing its clinical application and mitigating the development of resistance. Compared to conventional antibiotics, berberine poses a lower risk of triggering resistance. This is attributed to its multi-target antimicrobial mechanisms, which involve disrupting the cell membrane, interfering with DNA, and impairing energy metabolism (ImenshahiiI and Hosseinzadeh, 2019). Such complexity makes it challenging for bacteria to develop resistance through single mutations. Nevertheless, prolonged exposure to berberine may still drive adaptive evolution in bacteria, leading to resistance (Xia et al., 2022). By inducing berberine resistance in Escherichia coli, researchers can explore bacterial adaptation mechanisms under multifaceted stress, offering novel strategies to combat antibiotic resistance. Berberine disrupts biofilm integrity by inhibiting extracellular polymeric substance (EPS) synthesis and interfering with the quorum sensing (QS) system. Inducing berberine resistance in *E. coli* enables the study of the interplay between biofilm formation and drug resistance, laying a theoretical foundation for developing innovative anti-biofilm therapies. To further elucidate the mechanisms underlying *E. coli* resistance to TCM compounds, this study employs berberine monomer as the experimental agent. The use of a single-component substance eliminates confounding effects from impurities, ensuring clearer interpretation of experimental results. Additionally, berberine is a primary active ingredient in TCM herbs such as Scutellaria baicalensis (huangqin) and Coptis chinensis, both of which are renowned antibacterial agents in TCM. Thus, utilizing berberine to induce resistance in *E. coli* enhances the scientific rigor and relevance of this research.

To investigate whether Escherichia coli develops resistance to berberine and elucidate the underlying mechanisms, this study first induces berberine resistance in the E. coli ATCC25922 strain. We compare changes in biofilm formation before and after resistance induction, measure the mRNA levels of resistance-associated genes, and identify the key gene csgD. Subsequently, we construct a csgD-overexpressing strain and evaluate changes in its minimum inhibitory concentration (MIC), biofilm formation, and expression of biofilm-associated genes.

## 2 Materials and methods

### 2.1 Experimental materials

#### 2.1.1 Bacterial strains and plasmids

*E. coli* ATCC25922 strain was standard strain and DH5α were kept in our laboratory. The pTrc99a plasmid was purchased from BioCorp. The berberine induced strain was recorded as ATCC25922HLSYD, *csgD*-overexpressed strain was recorded as ATCC25922-csgD (OE), The empty plasmid strain was recorded as ATCC25922-pTrc99a.

#### 2.1.2 Reagents

Berberine hydrochloride HB8174 was purchased from Hefei Bomei Biological Company, with purity more than 98%. The dimethyl sulfoxide (DMSO) was purchased from Sobolai Biological Company. The methanol, crystal violet, and glacial acetic acid was purchased from Legend Biological Company. Tryptone, yeast extract, sodium chloride, and agar were purchased from Shenggong Biological Company. Sterile DEPC water, anhydrous ethanol, chloroform, isopropanol, Trizol, reverse transcription kit, and 2xSYBRqPCRmix were purchased from Novozymes Bio. Plasmid mini-extraction kit, gel recovery kit, ampicillin, T4DNA ligase, EcoRI restriction endonuclease, and XbaI restriction endonuclease were purchased from Novozymes Bio.

#### 2.1.3 Websites and software

NCBI (https://www.ncbi.nlm.nih.gov), primer5.0, GraphPadPrism9, SnapGene, Espript3 (https://espript.ibcp.fr), Clustal (https://www.genome.jp/tools-bin/clustalw), and SPSS 21.0 were used in our research.

#### 2.1.4 Primer sequences used in this study

The primer sequences used in this study are shown in **Table 1**.

**Table 1:** Primer names and sequences.

Primer name	Primer sequence (5’-3’)
*csgD*-qF	ATCGCTCGTTCGTTGTTC
*csgD*-qR	TCGCCTGAGGTTATCGTTT
*acrA*-qF	TCGCAGAAGTTCGTCCTC
*acrA*-qR	ACCTTTCGCACTGTCGTAT
*tolC*-qF	GTCACTTACCGACTCTGGAT
*tolC*-qR	GCGGAAACTACGGCTTGT
*bcsA*-qF	GCGGGCTTATTCTGCTCT
*bcsA*-qR	TGATGTTGCCTGCTTTCG
*luxS*-qF	TGCGTGCCGAACAAAGAA
*luxS*-qR	CAGCCCATTGGCGAGATA
*ompF*-qF	CGGTTATGGTCAGTGGGA
*ompF*-qR	GAGCTTCTTGCAGGTTGG
*ompC*-qF	TACGGCGTTGTTTATGAC
*ompC*-qR	ATGTAAGCAGCGGTGTTC
*acrB*-qF	GAACTACGACATCATCGCAGAG
*acrB*-qR	GCGTCATCCACCAACAGG
*csgD*-F	ATGTTTAATGAAGTCCATAG
*csgD*-R	TTATCGCCTGAGGTTATCGT
*csgD*-OEF	CCGGAATTCCGGATGTTTAATGAAGTCCATAG
*csgD*-OER	GCTCTAGAGCTTATCGCCTGAGGTT
Ptrc99a-F	CATCCGGCTCGTATAATGTGTG

### 2.2 Methods

#### 2.2.1 Determination of minimum inhibitory concentration

(1) Pick a single colony in Mueller-Hinton (MH) medium for 6–8 hours, use a pipette to suck up 200uL bacterial solution into 96-well plate, three parallel for each bacterium, and use an enzyme marker to measure the value at OD_630_nm between 0.35-0.4, at this time, the concentration of bacterial solution is 10^7–^10^8^ CFU/mL. (2) Dilute the bacterial solution with good OD value by 2000 times, and divide it into three dilution steps, the first step: Dilute 10 times that 100 μ L bacterial solution into 900 μ L MH medium; Step 2: Dilute 2 times that 100 μ L first step of the dilution of the bacterial solution into 100 μ L MH medium; Step 3: Dilute 100 times that 100 μ L second step of the dilution of the bacterial solution into the 10 mL MH medium mixing standby, this time the concentration of bacterial solution for the 5 × (10^3^-10^4^) CFU/mL. (3) Weigh the berberine dissolved into 10 mg/mL concentration with DMSO(DMSO concentration <0.1%), and dilute the berberine solution 20 times with methanol to obtain 500ug/mL of the berberine mother solution, and dilute the berberine mother solution 10 concentration gradients with the multiplicative dilution method, and the concentration of the berberine solution at this time was 500 μg/mL, 250 μg/mL, and 125 μg/mL, in that order, 62.5 μg/mL, 31.25 μg/mL, 15.625 μg/mL, 7.8125 μg/mL, 3.9 μg/mL, 1.95 μg/mL, 0.9765 μg/mL(4) Take a 96-well plate and start from the second row of the second wells and add 20uL of concentration gradient berberine solution and 80 μL of diluted bacterial solution sequentially until the 11th well. The concentration of berberine solution was 100 μg/mL, 50 μg/mL, 25 μg/mL, 12.5 μg/mL, 6.25 μg/mL, 3.125 μg/mL, 1.56 μg/mL, 0.78 μg/mL, 0.39 μg/mL, and 3.125 μg/mL, 1.56 μg/mL, 0.78 μg/mL, and 0.39 μg/mL, respectively, 0.195 μg/mL; in addition, 20 μL of berberine at different concentrations and 80 μL of LB medium were sequentially added to the first and last columns of the 96-well plate to serve as blank controls. 96-well plates were fixed in a shaker at 37°C and 200 rpm/min for 24 hours. (5) The MIC value was determined by the first clarified well before the turbid well. (6)The *E. coli* ATCC25922 was inoculated into Luria-Bertani (LB) medium containing berberine at a concentration of 1/2 MIC for overnight culture, with a total of 20 generations, The bacterial stock preserved in a -20°C freezer was inoculated into LB liquid medium at a 1:1000 ratio for overnight cultivation. The revived culture was then subcultured (1:1000 inoculation) in fresh LB medium containing berberine at 1/2 MIC concentration for overnight growth, with fresh medium replacement at each passage. Subculturing was performed every 12 hours for a total of 20 generations (10 days), while bacterial aliquots were preserved at each passage. The MIC value was determined every 4 generations throughout the experimental timeline. The *E. coli* was considered to be resistant to berberine when the MIC was ≥ 4-fold than that prior to induction. The berberine-induced strain was ATCC25922HLSYD (Yu et al., 2005; Song et al., 2020; George et al., 2025).

#### 2.2.2 Construction of *csgD-*overexpressed strain

The target gene *csgD* was amplified from ATCC25922 strain. The obtained *csgD* gene and pTrc99a plasmid were digested with EcoRI and XbaI (Novozymes Bio). After purified and ligated, the ligation products were transformed into DH5α receptor cells and cultured on ampicillin-resistant LB plates for overnight. The pTrc99a-csgD plasmid was obtained after validation by PCR and sequencing. pTrc99a-csgD plasmid was extracted and was transformed into ATCC25922 sensory with ampicillin-resistant LB plates for overnight. The overexpressed strain ATCC25922-csgD (OE) was obtained after verification by PCR and sequencing. ATCC25922-pTrc99a strain was also obtained by transforming pTrc99a plasmid into ATCC25922 sensory state as a control.

#### 2.2.3 Biofilm formation assay

The strains of standard, induced, and mutant were cultured overnight at 37°C, 200 rpm/min, and the concentration of the bacterial solution was adjusted to (1-5)×10^6^ CFU/mL. 100 μ L of the bacterial solution was put in a 96-well plate, with six replicates, and LB culture solution was used as negative control with six replicates. And then bacterial was cultured at 37°C for 24 h,48 h and 72 h, respectively. At the end of culture, the bacterial solution was aspirated and washed once with saline to remove the planktonic bacteria. After drying, 10 g/L crystal violet staining solution were added into the plate for 17 min. Subsequently, the excess crystal violet staining solution were removed and washed with physiological saline for three times. After drying, 100 μ L of 30% glacial acetic acid solution was added in the plate for 20 min. The absorbance value at OD_492_ nm was detected (Grossman et al., 2021; Wani et al., 2025).

#### 2.2.4 Relative expression of mRNA for biofilm-related genes

(1) Primer design: The sequence characteristics of *csgD*, *bcsA*, *luxS*, *ompF*, *ompC*, *acrA*, *acrB*, *tolC* genes were analyzed on NCBI, and specific primers were designed by using primer5.0 software. (2) Extraction of E. coli RNA: a. Bacteria cultured overnight were centrifuged at 4°C, 10,000 rpm/min, for 2 min, and the bacterial body was collected, about 50 mg. b. 1 mL of Trizol was added, and the mixture was shaken and mixed well, and then the sample was incubated in a 65°C water bath for 5 min and an ice bath for 5 min, and 200 μL of pre-cooled chloroform was added, and then the sample was mixed and left to stand at room temperature for 10 min. 4°C. 12000rpm/min, centrifuge for 10min, take the supernatant aqueous phase, and transfer to a new EP tube. c. Add pre-cooled isopropanol (1mL) in the same volume as Trizol, mix upside down, and let it stand at minus 20°C for 10min, to accelerate RNA precipitation. 12000rpm/min, 4°C, centrifugation for 10min, and discard the supernatant. d. Add 1mL 75% of ethanol (diluted with DEPC water), blow and mix, suspend the precipitate. Centrifuge at 8000 rpm/min, 4°C for 5 min, discard the supernatant. e. Dry at room temperature for 10 min, add 50 μL of sterile DEPC water to dissolve the RNA precipitate. Measure the concentration of RNA and put it in negative 80°C refrigerator for storage. (3) Reverse transcription: adjust the concentration of RNA to 1uL containing 100ng; 20uL of total system, 1uL of Total RNA, 1uL of RT mix, 4uL of RT buffer, 14uL of DEPC water; put the added system into the PCR instrument, and set up the program as follows: 37°C, 2min, 50°C, 15min, 85°C, 2min to carry out the reaction. (4) Real-time fluorescence quantitative PCR (qPCR): Dilute the CDNA obtained by reverse transcription 10 times before use; total system 20uL, CDNA 1uL, upper primer 0.5uL, lower primer 0.5uL, 2xSYBRqPCRmix 10uL, ddH2O 8uL. add the sample and put it into the qPCR instrument, set up the program as follows Pre-denaturation 95°C, 2min, denaturation 95°C, 15sec, annealing 60°C, 30sec, extension 72°C, 30sec. 40 cycles. (5) qPCR data processing and statistical analysis: Calculate the ΔCt, ΔΔCt and 2^-(ΔΔCt)^ of each group according to the Ct value of the target gene. Calculation method: ΔCt (target gene) = Ct value (target gene) - Ct value (internal reference gene), ΔΔCt (sensitive group) = ΔΔCt (sensitive group) - ΔΔCt (sensitive group), ΔΔCt (drug-resistant group) = ΔΔCt (drug-resistant group) - ΔΔCt (sensitive group), and relative quantitative results of the target genes of the resistant group are 2^-(ΔΔCt)^, and the comparison of the expression of each gene was performed in the form of fold change (Leung and Chan, 2022; Verbeelen et al., 2022).

#### 2.2.5 Statistical methods

Data were statistically analyzed using GraphPad Prism 9 and SPSS 21.0 software. Measured data statistical indexes satisfying normal distribution were expressed as mean ± standard deviation (). Repeated-measures ANOVA was used to analyze the amount of biofilm production before and after the induction of resistance by berberine; paired-samples t-test was used for the expression of relevant resistance genes before and after the induction of resistance by berberine; and one-way ANOVA was used for the determination of the amount of biofilm production in the strains before and after the overexpression of the *csgD* gene and for the relative expression of mRNA of the biofilm genes, and the two-by-two comparison of the information of multiple groups was made by the LSD method. *P* < 0.05 was regarded as statistically significant difference.

#### 2.2.6 Determination of *E. coli* survival rate using CCK-8 assay

(1) The revived bacterial strains were cultured overnight at 37°C with 200 rpm shaking until reaching an OD_630_ nm of 0.35–0.4. The adjusted bacterial suspension was diluted 2000-fold to achieve a final concentration of (1–5) × 10^6^ CFU/mL. (2) A 96-well plate was loaded with 100 μL of diluted bacterial suspension per well, with six replicate wells per strain. Wells containing 100 μL of LB medium served as negative controls. The plate was incubated at 37°C for 24 h. (3) Following incubation, 10 μL of CCK-8 solution was added to each well, followed by an additional 30-minute incubation. (4) Absorbance was measured at OD_450_ nm using a microplate reader. (5) Data were processed and analyzed using Excel and GraphPad Prism. (6) Bacterial survival rate (%) was calculated as:


$$\begin{array}{c} \text{Survival rate}=[\left(\text{Negative control well}-\text{Blank well}\right)\\ /\left(\text{Test well}-\text{Blank well}\right)]\times 100\% \end{array}$$

(Formula adapted from the CCK-8 assay protocol manual)

## 3 Results

### 3.1 The MIC of berberine against *E. coli*

**Figure 1:**
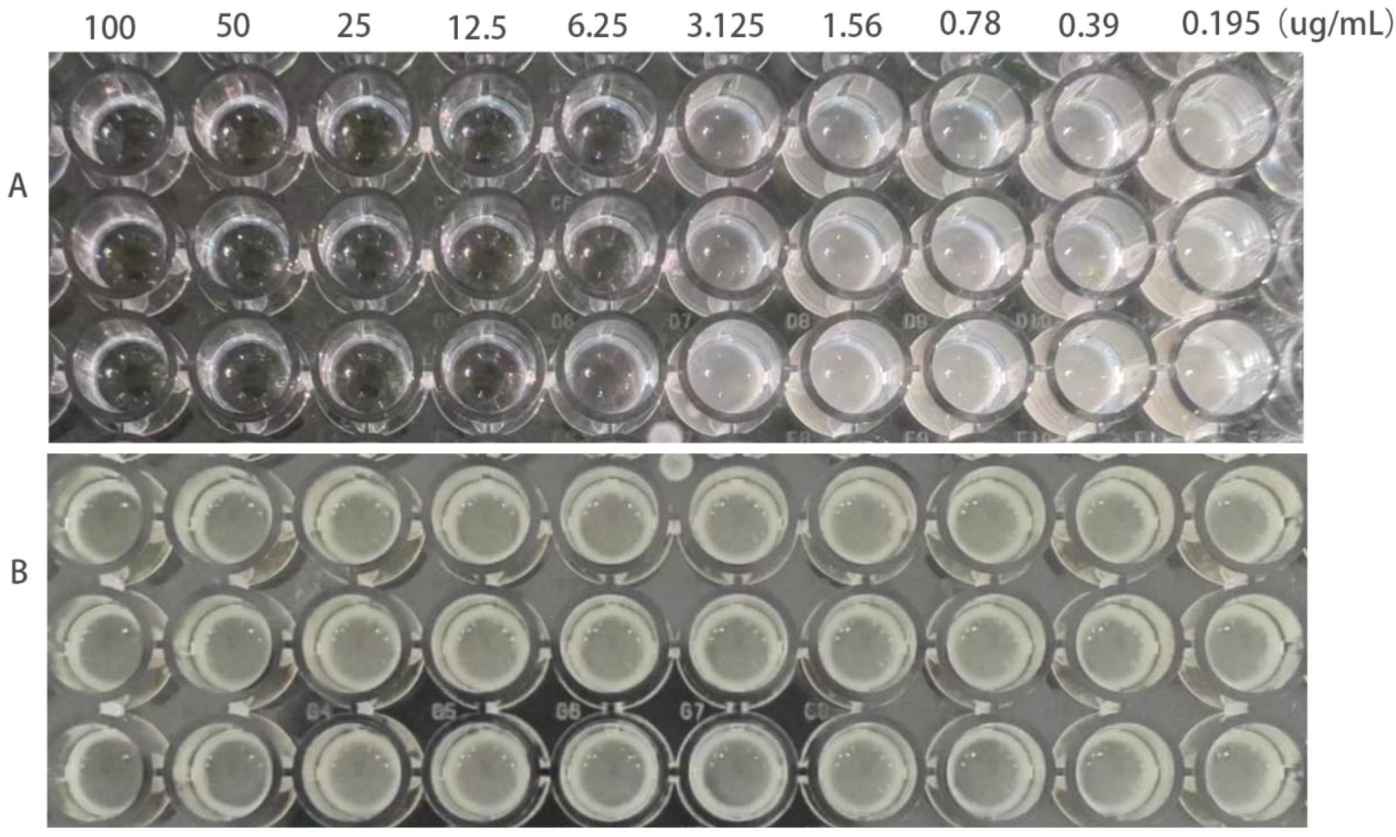
MIC values of *E. coli* before and after the induction of resistance by berberine. **(A)** refers to strain ATCC25922, **(B)** refers to strain ATCC25922HLSYD.

**Figure 2:**
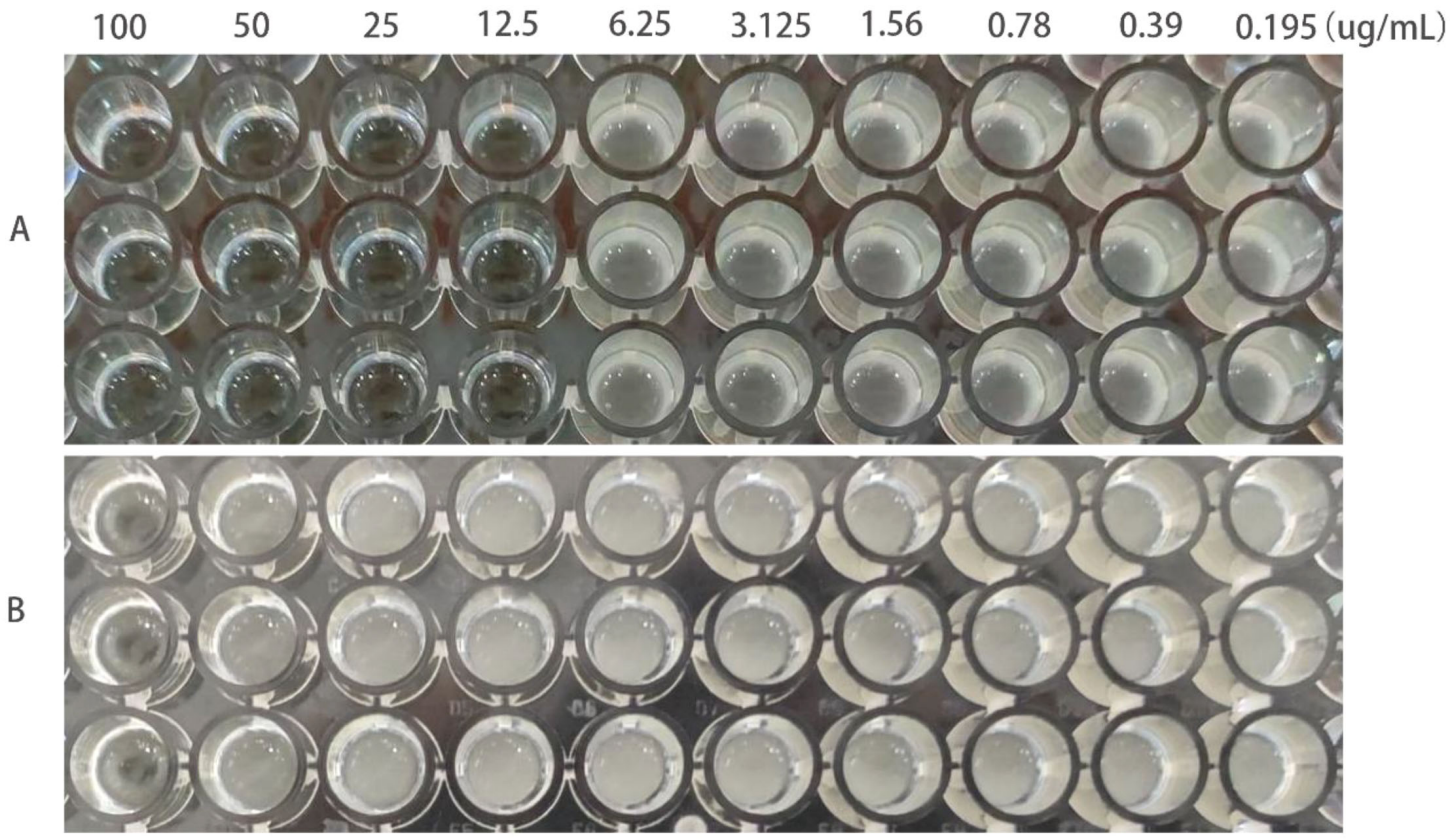
MIC values of berberine on ATCC25922-ptrc99a and ATCC25922-csgD (OE). **(A)** ATCC25922-ptrc99a strain; **(B)** ATCC25922-csgD (OE) overexpression strain.

The MIC of ATCC25922, ATCC25922HLSYD, ATCC25922-csgD (OE) and ATCC25922-pTrc99a were determined by two-fold dilution of trace broth, and the results were decided by the first clarified well before the turbid well, i.e. the MIC value measured. The results showed that the MIC of berberine against ATCC25922 was 6.25 μ g/mL and the 1/2MIC was 3.125 μ g/mL (**Figure 1A**). The resistance of berberine against ATCC25922 was induced by 1/2 MIC, the MIC after berberine induction was 200 μ g/mL (**Figure 1B**) with 32 times higher than that before induction. This result indicated that berberine-induced ATCC25922 resistance was successful. The relationship between specific induction generations and induction concentration was shown in **Table 2**. The MIC of the ATCC25922-csgD (OE) strain was 15-fold higher than that of the ATCC25922 strain (100 μ g/mL vs. 6.25 μ g/mL, **Figure 2B**). And the MIC of ATCC25922-pTrc99a strain was 12.5 μ g/mL with 1-fold higher than that of the ATCC25922 strain (**Figure 2A**). By comparing the MIC with ATCC25922-csgD (OE) strain and ATCC25922-pTrc99a strain, it indicated that the elevated MIC of the overexpression strain might result from the overexpression of *csgD*.

**Table 2:** Relationship between induced concentration and cumulative time and generations.

Induction cumulative time(h)	MIC(μg/mL)	Generation
0	3.125	0
48	6.25	4
96	12.5	8
144	25	12
192	50	16
240	100	20

### 3.2 Construction of ATCC25922-csgD (OE) overexpression strain

**Figure 3:**
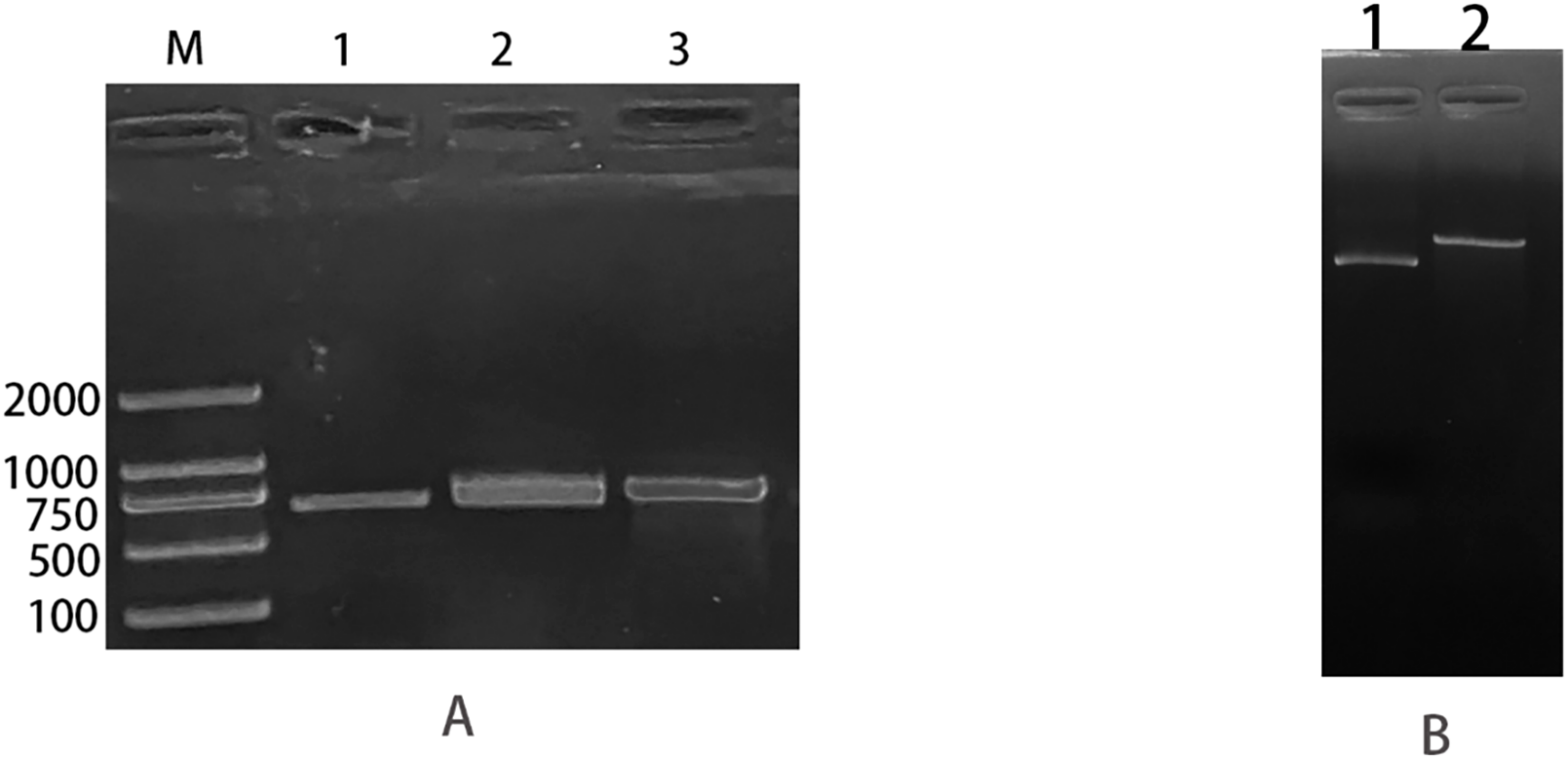
ATCC25922-csgD (OE) overexpression strain construction. **(A)** M: marker; 1: *csgD* target gene amplified using ATCC25922 as template, with a band size of 660 bp; 2: fragment size of 673 bp obtained from *csgD* target gene digested with EcoRI and XbaI endonuclease; 3: csgD-OER using pTrc99a-F, csgD-OER as primers, and the ATCC25922 -csgD (OE) overexpression strain as template, PCR amplified a cross fragment of about 750 bp. **(B)** 1: pTrc99a plasmid not digested; 2: fragment obtained by double digestion of PTrc99a plasmid with endonucleases EcoRI and XbaI.

**Figure 4:**
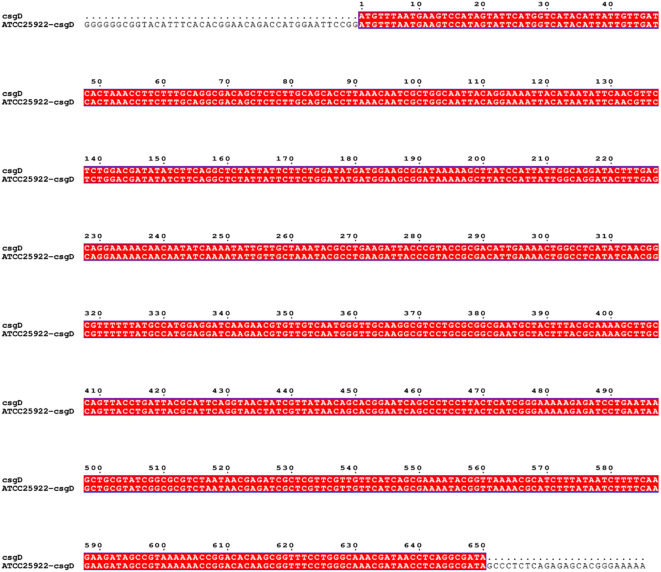
Sequence comparison of cross-fragment and *csgD*.

The pTrc99a plasmid and *csgD* gene were double digested and ligated to construct an overexpression vector, and the overexpression vector was transfected into TACC25922 receptor cells to construct an overexpression strain. The function of *csgD* gene was verified by constructing ATCC25922-csgD (OE) overexpression strain. Agarose electrophoresis gel results showed that the successfully digested *csgD* gene fragment was 672 bp (**Figure 3A**), the pTrc99a plasmid (**Figure 3B**), and the size of the identified fragment was 750 bp (**Figure 3A**). In addition, the identified fragment was completely identity with the sequence of the *csgD* gene by sequencing. (**Figure 4**), which indicated that the ATCC25922-csgD(OE) overexpression strain was successfully constructed.

### 3.3 Detection of biofilm formation in standard strain, induced, and mutant strains of bacteria

**Figure 5:**
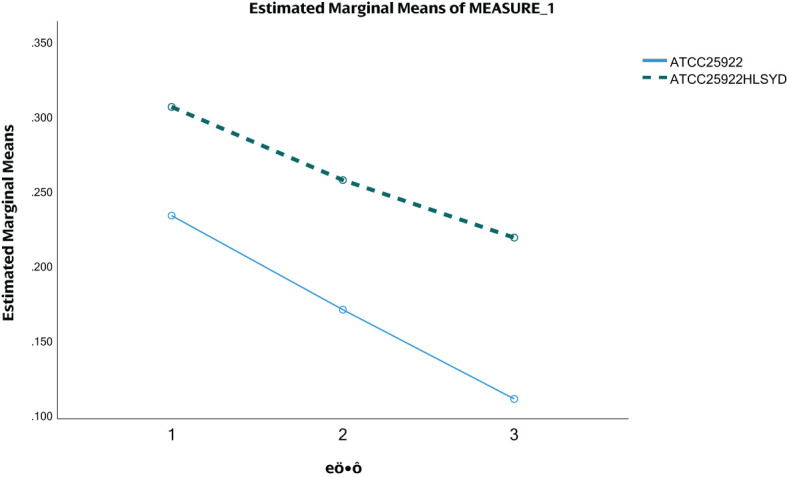
ATCC25922 and ATCC25922HLSYD biofilm formation. 1 for 24 hours, 2 for 48 hours and 3 for 72 hours.

**Figure 6:**
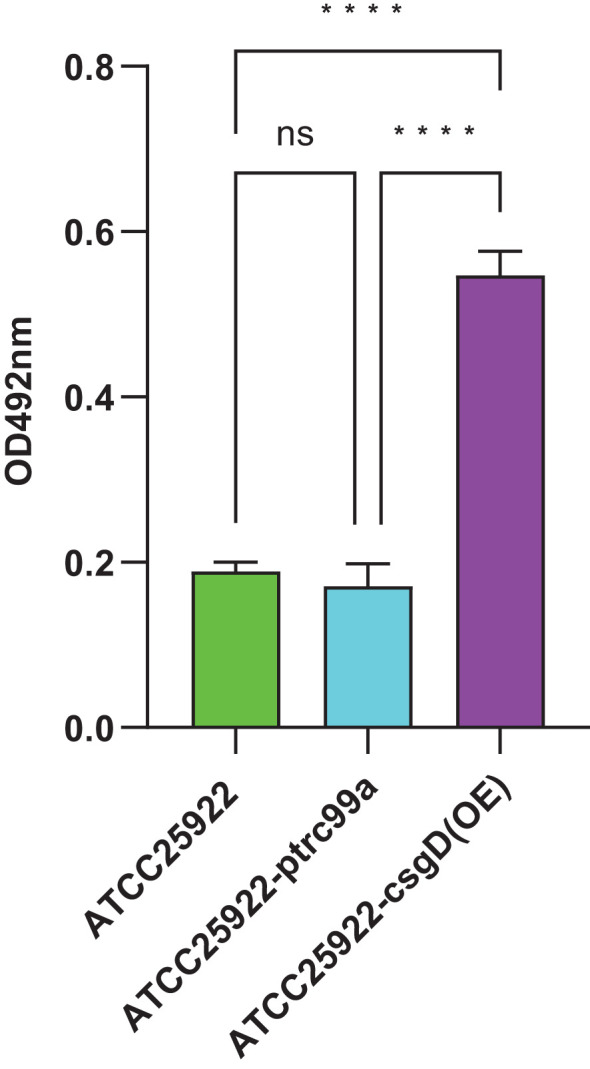
Measurement of biofilm formation of ATCC25922, ATCC25922-ptrc99a, and ATCC25922-csgD (OE). ns represents no significant difference, **** represents *P* < 0.001.

We tested ATCC25922, ATCC25922HLSYD, ATCC25922-pTrc99a and ATCC25922-csgD (OE) strains for biofilm formation, It turns out that the formation of biofilm in the berberine-induced resistant strain ATCC25922HLSYD was significantly increased (*p* < 0.001) compared to that of ATCC25922 (**Table 3**; **Figure 5**). In addition, the formation of biofilm was gradually decreased in a time-dependent manner (*p* < 0.001). There was no significant difference between time and group (**Table 3**; **Figure 5**). The formation of biofilm was significantly increased at 24, 48, and 72 hours after berberine induction, with 1.3-fold, 1.51-fold, and 1.98-fold higher than that before induction, respectively. The biofilm formation of ATCC25922-csgD (OE) was significantly increased compared to that of ATCC25922 (*p* < 0.001), which was 2.9 times higher than that of ATCC25922 (**Table 4**; **Figure 6**). The biofilm formation of ATCC25922-csgD (OE) was significantly increased compared to that of ATCC25922-pTrc99a (*p* < 0.001), which was 3.2 times higher than that of ATCC25922-pTrc99a (**Table 4**; **Figure 6**).

**Table 3:** Results of biofilm formation before and after the induction of resistance by berberine ( $\bar{x}\pm s)$ .

Groups	24 h	48 h	72 h	*F*	*P*
ATCC25922	0.233±0.022	0.170±0.015	0.111±0.037		
ATCC25922HLSYD	0.306±0.033	0.257±0.026	0.219±0.012		
time main effect				56.837	<0.001
Between-group main effects				89.213	<0.001
Time* between-group interaction effect				1.618	0.239

*p*<0.001 compared to 24 h and 48 h; *p*<0.001 compared to 24 h and 72 h; *p*=0.003 compared to 48 h and 72 h.

**Table 4:** Comparative results of biofilm formation of strains before and after overexpression of *csgD* gene ( $\bar{x}\pm s$ ).

Strains	Biofilm production	*F*	*P*
ATCC25922	0.189±0.011	307.464	<0.001
ATCC25922-pTrc99a	0.170±0.027		
ATCC25922-csgD(OE)	0.547±0.030		
ATCC25922 VS ATCC25922-pTrc99a	0.018±0.171		0.32
ATCC25922 VS ATCC25922- csgD(OE)	-0.358±0.171		<0.001
ATCC25922-pTrc99a VS ATCC25922- csgD(OE)	-0.376±0.171		<0.001

### 3.4 Detection of the expression of resistance-related genes standard strain, induced strain and mutant strain

**Figure 7:**
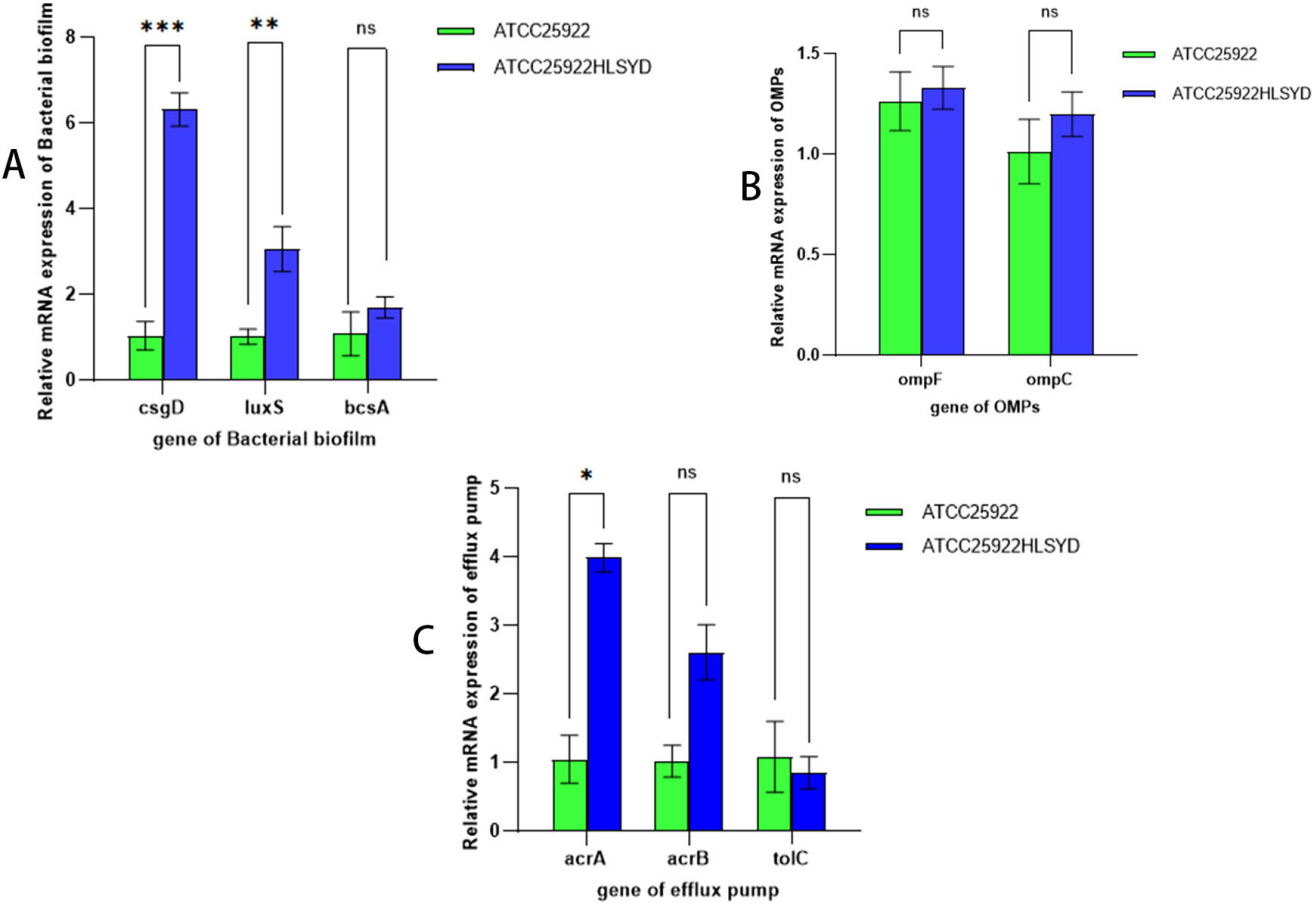
Relative expression of mRNAs of drug resistance-related genes before and after induction of resistance by berberine. **(A)** refers to biological periplasmic genes, **(B)** refers to membrane protein genes, and **(C)** refers to efflux pump genes; * stands for *P*=0.040; ** stands for *P*=0.037; ** stands for *P*=0.016; and ns stands for the difference is not statistically significant.

By examining the expression of resistance-related genes in strains ATCC25922 and ATCC25922HLSYD, we found that the expression of the biofilm related genes, *csgD* and *luxS*, was significantly increased in ATCC25922HLSYD strain (*p* < 0.05) (**Table 5**; **Figure 7A**). There was no significant difference of membrane protein-related genes *ompF* and *ompC* in ATCC25922 and ATCC25922HLSYD (**Table 5**; **Figure 7B**). The expression of efflux pump-related gene *acrA* was significantly increased in ATCC25922HLSYD (*p*=0.040), and the expression of *acrB* and *tolC* showed no significant change between ATCC25922 and ATCC25922HLSYD (**Table 5**; **Figure 7C**). And the expression of gene *csgD* showed the greatest change in ATCC25922and ATCC25922HLSYD csgD gene expression was 5.8 times higher than ATCC25922 (**Table 5**; **Figure 7A**). Therefore, the gene *csgD* was subsequently used as a key gene for further study.

**Table 5:** RT-qPCR results of drug resistance-related genes in strains before and after induction of resistance by berberine ( $\bar{x}\pm s$ ).

Genetics	Difference in expression before and after induction	*T*	*P*
*csgD*	-5.61±1.23	-7.928	0.016
*luxS*	-2.05±0.71	-5.020	0.037
*bcsA*	-1.62±1.50	-1.871	0.202
*ompF*	0.20±1.38	0.252	0.825
*ompC*	-0.19±0.74	-0.044	0.969
*acrA*	-3.04±1.09	-4.845	0.04
*acrB*	-1.78±1.22	-2.52	0.128
*tolC*	0.23±0.35	1.137	0.373

**Figure 8:**
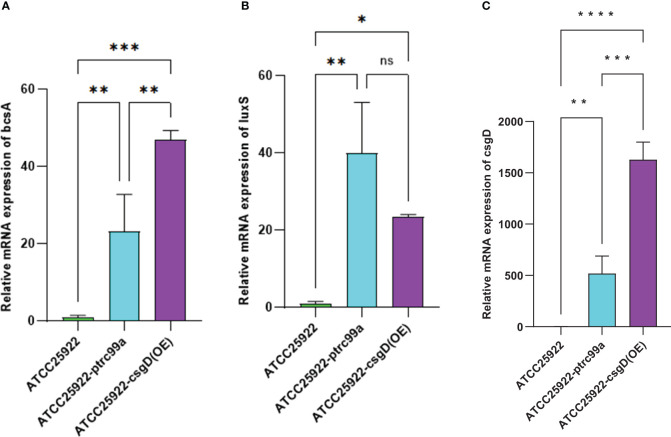
Relative expression of mRNAs of biofilm related genes in ATCC25922, ATCC25922-ptrc99a, and ATCC25922-csgD (OE). **(A)** refers to *bcsA* gene; **(B)** refers to *luxS* gene; **(C)** refers to *csgD* gene; * stands for *P* < 0.05, ** stands for *P* < 0.01, ***stands for *P* < 0.005, **** stands for *P* < 0.001, and ns stands for no significant difference.

By examining the expression of biofilm-associated genes in strains ATCC25922, ATCC25922-pTrc99a, and ATCC25922-csgD(OE), we found that the expression of the *bcsA* gene in the ATCC25922-csgD (OE) strain was significantly increased compared to that of the ATCC25922 strain (*p*<0.001), which was 45-fold higher than that of the ATCC25922 strain. And the expression of *bcsA* gene was significantly increased in ATCC25922-csgD (OE) strain compared to ATCC25922-pTrc99a strain(*p*=0.002), which was 2-fold higher than ATCC25922-pTrc99a, (**Table 6**; **Figure 8A**). The *luxS* gene expression was significantly increased compared to ATCC25922 strain in ATCC25922-pTrc99a (*p*=0.012), with 22.5-fold than that of ATCC25922 strain (**Table 7**; **Figure 8B**). The expression of *luxS* gene in ATCC25922-csgD (OE) strain was not significantly different from that of ATCC25922-pTrc99a strain (**Table 7**; **Figure 8B**). The expression of *csgD* gene in ATCC25922-csgD (OE) strain was significantly increased (*p*<0.001), with 1628 times higher than that in ATCC25922 strain. In addition, the expression of *csgD* gene in ATCC25922-csgD (OE) strain was significantly increased (*p*<0.001), which was 3.13 times higher than that in ATCC25922-ptrc99a strain (**Table 8**; **Figure 8C**).

**Table 6:** The mRNA expression of *bcsA* gene in strains before and after overexpression of *csgD* gene ( $\bar{x}\pm s$ ).

Strains	*bcsA* gene mRNA expression	*F*	*P*
ATCC25922	1.041±0.210	49.254	<0.001
ATCC25922-ptrc99a	23.227±5.470		
ATCC25922-csgD(OE)	46.870±1.423		
ATCC25922 VS ATCC25922-ptrc99a	-22.185±4.618		0.003
ATCC25922 VS ATCC25922- csgD(OE)	-45.829±4.618		<0.001
ATCC25922-ptrc99aVSATCC25922-csgD(OE)	-23.644±4.618		0.002

**Table 7:** The mRNA expression of *luxS* gene in strains before and after overexpression of *csgD* gene ( $\bar{x}\pm s$ ).

Strains	*luxS* gene mRNA expression	*F*	*P*
ATCC25922	1.050±0.232	19.55	0.002
ATCC25922-ptrc99a	39.892±7.624		
ATCC25922-csgD(OE)	23.360±0.365		
ATCC25922 VS ATCC25922-ptrc99a	-38.842±6.235		0.001
ATCC25922 VS ATCC25922- csgD(OE)	-22.311±6.235		0.012
ATCC25922-ptrc99a VS ATCC25922- csgD(OE)	16.532±6.235		0.038

**Table 8:** The mRNA expression of *csgD* gene in strains before and after *csgD* gene overexpression ( $\bar{x}\pm s$ ).

Strains	*csgD* gene mRNA expression	*F*	*P*
ATCC25922	1.163±0.370	107.459	<0.001
ATCC25922-ptrc99a	519.943±97.433		
ATCC25922-csgD(OE)	1628.042±98.93		
ATCC25922 VS ATCC25922-ptrc99a	-518.779±113.374		0.004
ATCC25922 VS ATCC25922- csgD(OE)	-1626.878±113.374		<0.001
ATCC25922-ptrc99a VS ATCC25922- csgD(OE)	-1108.099±113.374		<0.001

### 3.5 Survival rate results of *E. coli*

**Figure 9:**
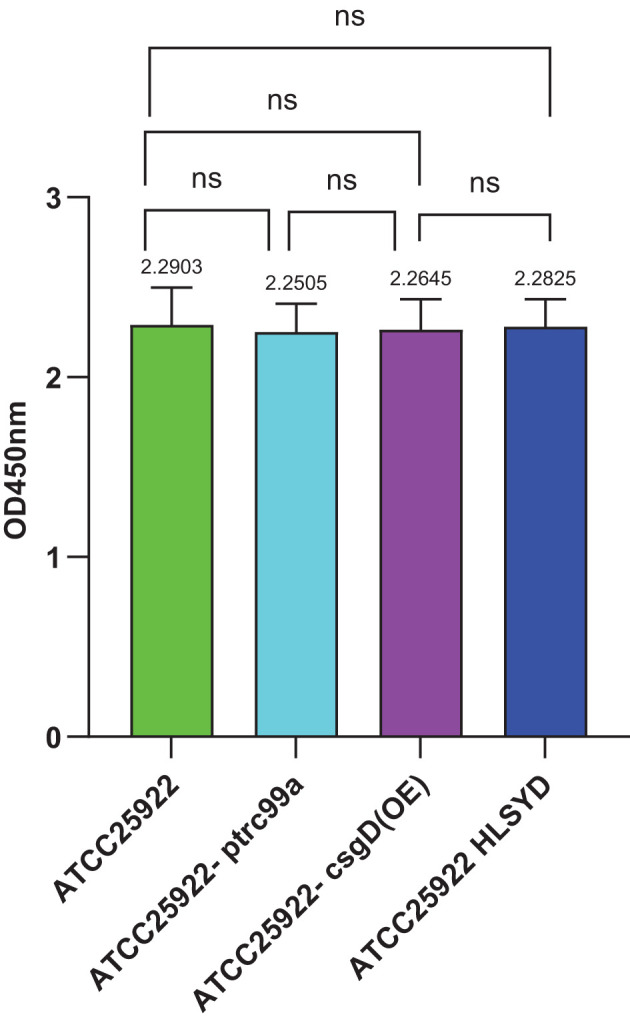
CCK-8 staining of Escherichia coli strains ATCC25922, ATCC25922-ptrc99a, ATCC25922-csgD (OE), ATCC25922HLSYD. ns represents no significant difference.

The CCK-8 assay was performed to evaluate the viable bacterial counts of *E. col* strains ATCC25922, ATCC25922-ptrc99a, ATCC25922-csgD(OE), and ATCC25922HLSYD. The results revealed no statistically significant differences in survival rates among these strains (**Figure 9**), indicating that biofilm formation is independent of the quantity of non-viable bacterial cells.

## 4 Discussion

In this study, we induced Escherichia coli with a sub-minimum inhibitory concentration (sub-MIC, 3.125 μ g/mL) of berberine for 20 consecutive generations, totaling 240 hours. We observed that the minimum inhibitory concentration (MIC) value significantly increased to over 100 μ g/mL after induction, which was more than 32 times higher than the pre-induction value. This result stands in stark contrast to the findings of Yuhui Zhan (2020) et al. In their study, *E. coli* was induced with a sub-MIC of 8 μ g/mL amikacin for a total of 297 hours, resulting in an MIC value of 256 μ g/m L. Although the induction concentration of amikacin was higher, the increase in MIC value post-induction was relatively smaller, suggesting that berberine is more likely to induce resistance in *E. coli* compared to amikacin. This phenomenon may be related to the multi-target mechanism of berberine. Despite its broad-spectrum activity, prolonged exposure may still lead to bacterial resistance through adaptive evolution.

Further research revealed that after berberine-induced resistance, the biofilm formation ability of E. coli significantly increased at 24, 48, and 72 hours, with biofilm production being 1.3-1.98 times higher than before induction. This result is consistent with the findings of Cui Jiuhong and Gong Luyao (Cui and Tong, 2016; Gong, 2017), indicating that the resistance of *E. coli* to berberine is closely related to biofilm formation. Notably, the increase in biofilm formation was most pronounced at 72 hours, a time point that coincides with the transition from biofilm maturation to dispersal. This suggests that berberine may enhance bacterial resistance by prolonging the process from biofilm maturation to dispersal. The biofilm formation process includes four stages: initial adhesion, microcolony formation, maturation, and dispersal (Babushkina et al., 2020). At 72 hours, the biofilm is in the late maturation stage, and bacteria may enhance biofilm stability to resist the antibacterial effects of berberine.

To further explore the molecular mechanisms of berberine-induced resistance, we measured the expression levels of resistance-related genes before and after induction. The results showed that the expression of the *csgD* gene significantly increased (p=0.016), reaching 5.8 times the pre-induction level, making it the most significantly changed gene among those related to biofilm formation. The *csgD* gene encodes a transcriptional regulatory protein that plays a key role in biofilm formation, and its significant upregulation suggests that the resistance of *E. coli* to berberine may be closely related to the regulation of the *csgD* gene. Additionally, we found that the expression levels of the *luxS* and *bcsA* genes increased by 22.5 times and 45 times, respectively. The *luxS* gene is involved in the regulation of the quorum sensing system, while the *bcsA* gene encodes cellulose synthase, both of which are closely related to biofilm formation. These results indicate that the *csgD* gene may positively regulate the expression of *luxS* and *bcsA* genes, promoting the secretion of extracellular polymers (such as cellulose) and the activation of the quorum sensing system, thereby enhancing biofilm formation.

To validate this hypothesis, we constructed an E. coli strain overexpressing the *csgD* gene and measured the MIC value of berberine against this strain. The results showed that the MIC value of berberine for the csgD-overexpressing strain increased by 16 times, while biofilm production increased by 2.9 times. Furthermore, the expression levels of *luxS* and *bcsA* genes increased by 22.5 times and 45 times, respectively. These results further confirm the critical role of the *csgD* gene in regulating biofilm formation and resistance. Consistent with the findings of Chen Huan (Chen et al., 2023) et al., overexpression of the *csgD* gene not only promoted the secretion of extracellular polymers and bacterial motility but also enhanced the activity of the quorum sensing system, significantly improving biofilm formation ability.

Although the biofilm biomass of the overexpressing strain [ATCC25922-csgD(OE)] increased by 2.9-fold compared to the wild-type control—significantly higher than the 1.98-fold increase in the induced strain (ATCC25922HLSYD) relative to its pre-induction state—its MIC increase (16-fold) was lower than that of the induced strain (32-fold). This suggests that biofilm biomass alone may not fully account for berberine resistance. In the induced strain (ATCC25922HLSYD), the most significant biofilm increase occurred at 72 h (1.98-fold), coinciding with the critical transition phase from biofilm maturation to dispersal (**Figure 5**). This implies that berberine may enhance biofilm stability and barrier function by prolonging the maturation stage. We note that the lower MIC increase in the overexpressing strain [ATCC25922-csgD(OE)]—despite its higher biomass—may indicate the involvement of non-biofilm mechanisms in the induced strain (ATCC25922HLSYD). Given berberine’s multi-target properties (e.g., disruption of membrane integrity and DNA interference), prolonged induction (20 generations, 240 h) could trigger additional adaptive changes, such as efflux pump activation or metabolic dormancy. Future studies should investigate these potential co-mechanisms.

In summary, this study reveals the potential mechanisms of berberine-induced resistance in *E. coli*. Prolonged exposure to berberine may lead to significant upregulation of the *csgD* gene, which in turn positively regulates the expression of *luxS* and *bcsA* genes, promoting biofilm formation and stability. Biofilm formation not only provides a physical barrier for bacteria but also enhances bacterial tolerance to antibiotics by prolonging the process from biofilm maturation to dispersal. This discovery provides new insights into the mechanisms of berberine resistance and highlights the need to be cautious about the potential risk of resistance when using berberine as an antibacterial agent. Future research could further explore the specific mechanisms of the *csgD* gene regulatory network and how to overcome bacterial resistance by targeting the *csgD* gene or its downstream pathways.

## 5 Conclusions

1. Berberine significantly increased the minimum inhibitory concentration (MIC) of Escherichia coli, with the post-induction MIC value exceeding 100 μg/mL, which was more than 32 times higher than the pre-induction value. Compared to amikacin, berberine was more likely to induce resistance in E. coli. Although berberine exhibits broad-spectrum antibacterial activity, prolonged exposure may still lead to bacterial resistance through adaptive evolution.
2. Further research revealed that After berberine-induced resistance, the biofilm formation ability of E. coli was significantly enhanced, particularly at 72 hours, where biofilm production reached 1.3-1.98 times the pre-induction level. This suggests that berberine may enhance bacterial resistance by prolonging the transition from biofilm maturation to dispersal. Biofilm formation not only provides a physical barrier for bacteria but also enhances biofilm stability, helping bacteria resist the antibacterial effects of berberine.
3. At the molecular level, the expression of the *csgD* gene significantly increased (*p*=0.016), reaching 5.8 times the pre-induction level, indicating its critical role in regulating biofilm formation and resistance. Additionally, the expression levels of the *luxS* and *bcsA* genes increased by 22.5 times and 45 times, respectively, further confirming that the *csgD* gene positively regulates the quorum sensing system and cellulose synthase expression, promoting the secretion of extracellular polymers and biofilm formation. By constructing an E. coli strain overexpressing the *csgD* gene, we found that the MIC value of berberine increased by 16 times, biofilm production increased by 2.9 times, and the expression levels of *luxS* and *bcsA* genes were also significantly upregulated. These results further validate the central role of the *csgD* gene in regulating biofilm formation and resistance.

## Data Availability

The original contributions presented in the study are included in the article/**Supplementary Material**. Further inquiries can be directed to the corresponding authors.
